# Disability and recovery in schizophrenia: a systematic review of cognitive behavioral therapy interventions

**DOI:** 10.1186/s12888-016-0912-8

**Published:** 2016-07-11

**Authors:** Izabela Nowak, Carla Sabariego, Piotr Świtaj, Marta Anczewska

**Affiliations:** First Department of Psychiatry, Institute of Psychiatry and Neurology, Sobieskiego 9, 02-957 Warsaw, Poland; Department of Medical Informatics, Biometry and Epidemiology – IBE, Chair for Public Health and Health Services Research, Research Unit for Biopsychosocial Health, Ludwig-Maximilians-Universität München, Munich, Germany

**Keywords:** Schizophrenia, Disability, International Classification of Functioning, Disability and Health (ICF), Personal recovery, Cognitive-behavioral therapy

## Abstract

**Background:**

Schizophrenia is a disabling disease that impacts all major life areas. There is a growing need for meeting the challenge of disability from a perspective that extends symptomatic reduction. Therefore, this study aimed to systematically review the extent to which traditional and “third wave” cognitive – behavioral (CBT) interventions address the whole scope of disabilities experienced by people with lived experience of schizophrenia using the WHO’s International Classification of Functioning, Disability and Health (ICF) as a frame of reference. It also explores if current CBT interventions focus on recovery and what is their impact on disability domains.

**Methods:**

Medline and PsycINFO databases were searched for studies published in English between January 2009 and December 2015. Abstracts and full papers were screened against pre-defined selection criteria by two reviewers. Methodological quality of included studies was assessed by two independent raters using the Effective Public Health Practice Project Quality assessment tool for quantitative studies (EPHPP) guidelines.

**Results:**

A total of 50 studies were included, 35 studies evaluating traditional CBT interventions and 15 evaluating “third wave” approaches. Overall, traditional CBT interventions addressed more disability domains than “third wave” approaches and mostly focused on mental functions reflecting schizophrenia psychopathology. Seven studies met the inclusion criteria of recovery-oriented interventions. The majority of studies evaluating these interventions had however a high risk of bias, therefore evidence on their effectiveness is inconclusive.

**Conclusions:**

Traditional CBT interventions address more disability domains than “third wave” therapies, however both approaches focus mostly on mental functions that reflect schizophrenia psychopathology. There are also few interventions that focus on recovery. These results indicate that CBT interventions going beyond symptom reduction are still needed. Recovery-focused CBT interventions seem to be a promising treatment approach as they target disability from a broader perspective including activity and participation domains. Although their effectiveness is inconclusive, they reflect users’ views of recovery and trends towards improvement of mood, negative symptoms and functioning are shown.

**Electronic supplementary material:**

The online version of this article (doi:10.1186/s12888-016-0912-8) contains supplementary material, which is available to authorized users.

## Background

Schizophrenia is a psychotic disorder that affects about 26 million people around the world [1]. It is typically diagnosed in early adulthood, and mostly persists throughout peoples’ lives [2]. Several long-term follow-up studies have challenged the view about schizophrenia poor outcome and proved that varying degrees of improvement are possible [3–5], however schizophrenia is still one of the main causes of disability worldwide [6].

The World Health Organization International Classification of Functioning, Disability and Health – ICF [7] provides a unified and standard language for the description of health, functioning and disability. According to the ICF disability encompasses impairments of body functions, activity limitations or participation restrictions, arising as a result of interaction between a health condition and contextual factors (i.e. personal and environmental factors). An individual with lived experience of schizophrenia, might experience disability due to impairments of thought or perceptual functions (mental functions) as well as difficulties in family relationships and interpersonal interactions or in acquiring and keeping a job (activities and participation). Environmental factors such as accessibility to health services and appropriate treatment, systems and policies of a country, or stigmatizing societal attitudes as well as personal factors e.g. low self-esteem impact the level of disability experienced.

Results of a systematic review by Świtaj et al. [8] indeed revealed that disability in schizophrenia does not only refer to impairments of mental functions but also to several activities and participation domains. This is in line with the recently developed ICF Core Set for Schizophrenia, which in addition to the above mentioned domains also includes various environmental factors [9]. As a result, there has been a growing need on the part of clinicians and researchers to meet the challenge of reducing disability faced by people with schizophrenia from a broader perspective.

Key elements of the broader perspective are service users’ views on personal recovery understood as the achievement of a valued, meaningful life, rather than clinical recovery [10, 11]. Elements of personal recovery have been recaptured in the systematic review carried out by Leamy et al. [12] who identified them as: connectedness; hope and optimism about the future; identity; meaning in life; and empowerment (giving the acronym CHIME). Lloyd et al. [13] revealed that subjective indicators of recovery such as empowerment are associated with more objective recovery dimensions such as level of participation in the community and income from employment.

From the perspective of treatment various forms of psychosocial interventions have been recommended as an adjunct to medications, with cognitive-behavioral therapy (CBT) being one of the acknowledged interventions [14, 15]. Cognitive behavioral therapy for psychosis (CBTp) has gained large empirical support, however results obtained in meta-analytic reviews [16–19], have been mixed leading to a debate over the effectiveness of CBTp and pointing the need of its further development. One way of improving the outcomes of traditional CBT approaches is the inclusion of “third wave” CBT or more recently called “contextual” approaches to the theory and practice. Traditional CBT interventions actively focus on identifying and changing the thought content in specific disorders, whereas “third wave” approaches focus on modifying the context and function of thoughts [20]. A number of interventions represent “third wave” oriented CBT e.g. mindfulness-based cognitive therapy (MBCT) [21], metacognitive therapy (MCT) [22], acceptance and commitment therapy (ACT) [23], dialectical behavior therapy (DBT) [24], functional analytic psychotherapy (FAP) [25], integrative behavioral couple therapy (IBCT) [26] or person-based cognitive therapy for psychosis [27]. There is a growing evidence supporting “third wave” interventions in schizophrenia indicating moderate effect sizes in reducing negative symptoms [28].

Current systematic reviews on CBT interventions in schizophrenia mostly focus on symptomatic recovery, i.e. on mental functions, as a primary treatment target [18, 19]. Although symptom reduction is undeniably important in schizophrenia it is less clear the extent to which traditional and “third wave” CBT interventions address the whole scope of disabilities experienced by people with lived experience of schizophrenia. Considering service users definitions of recovery it is also important to explore, whether current CBT interventions focus on recovery and what is their impact on disability domains. This information would be useful in the development of CBT interventions that aim to support people with lived experience of schizophrenia in overcoming disabilities experienced in daily life through a process that is in line with the personal recovery approach. The objective of this systematic review is therefore threefold:

1. to provide a comprehensive overview on disability domains considered by traditional and “third wave” CBT interventions in schizophrenia using the ICF as a frame of reference;
2. to examine whether there are CBT interventions focusing on personal recovery;
3. to examine if recovery-oriented interventions effectively impact targeted disability domains.

The review specifically answers the following questions:

- which disability domains are being addressed by current CBT approaches?
- are there CBT interventions that focus on recovery?
- what is the effectiveness of CBT interventions focusing on recovery?

## Methods

We adhered to the PRISMA guidelines for conducting and reporting systematic reviews for evaluating health care interventions [29].

### Search strategy

A systematic search was conducted using Medline and PsycINFO databases. Keywords, MeSH and Index terms for the search strategy were identified through reviewing systematic reviews [16, 30] and studies relevant to the pre-established diagnosis, intervention and study design. The full search strategy is presented in Additional file 1. The reference lists of previously published reviews were also screened in order to identify papers that might have been omitted in the systematic search.

### Selection criteria

Studies were included if:

- published in English between January 2009 and December 2015,
- considered adults (18–65 years), at least 50 % of the sample under the study with schizophrenia spectrum disorders diagnosed by the International Classification of Diseases, tenth edition (ICD-10) [31] or Diagnostic and Statistical Manual of Mental Disorders, fourth edition (DSM-IV) [32]. We also included studies in which schizophrenia diagnosis was confirmed on the basis of medical records or the diagnostic criteria have not been explicitly specified but the study considered people with spectrum of schizophrenia disorders.
- study design was Randomized Controlled Trial (RCT), Clinical Controlled Trial (CCT), observational study with/without control group,
- schizophrenia disability dimension was an outcome,
- referred to traditional or “third wave” CBT interventions.

With regards to traditional approaches we selected studies with intervention description including reference to (a) establishing links between outcomes, thoughts and beliefs, distress or problem behavior (b) included re-evaluation of perceptions, beliefs or reasoning [16] relating to the target outcomes. Regarding “third wave” interventions we included all acceptance-based, compassion-based, and mindfulness-based approaches and interventions using “third wave” strategies as one of the central components of the treatment conceptualization. In order to assess whether interventions focused on recovery, we set the following criteria: indication by authors that the intervention refers to recovery, which is further linked to personal recovery oriented intervention formulation or inclusion of personal recovery aspects in the intervention conceptualization. Personal recovery was defined according the CHIME framework [12].

Studies were excluded if:

- included participants with active drug or alcohol dependence, organic brain disease, severe cognitive deficits or documented mental retardation,
- considered primary prevention studies, phase I and II study, ecologic studies, case reports, case series, cross-sectional studies, qualitative studies, economic evaluations,
- the primary target of interventions was not effectiveness,
- CBT forming part of broader interventions,
- hybrid forms of existing cognitive therapy,
- considered unpublished studies, book chapters, dissertations, commentaries, letters to the editors, editorials, conference reports.

### Eligibility assessment

Abstracts retrieved from databases were examined against the selection criteria by a trained reviewer. To increase reliability of this process 20 % of randomly selected abstracts were double checked by a second reviewer, who was blind to the decision of the first researcher. Papers considered eligible were retrieved and examined by two researchers.

### Data extraction and data synthesis

One reviewer extracted the following data from the included papers: the objectives of the study; study design; study population; outcome variables and questionnaires used; disability aspects; recovery orientation; and results. Extracted information about interventions involved the name, number of sessions, duration and frequency, intervention description and manual used. A matrix of traditional and “third wave” CBT interventions was provided with regards to conceptually or thematically-related categories of disability based on the ICF using the linking rules described by Cieza et al. [33]. A separate categorization of interventions focusing on recovery was also performed.

### Methodological assessment

Included studies were independently assessed by two researchers using the Effective Public Health Practice Project (EPHPP) quality assessment tool [34]. This instrument permits quality evaluation of a wide range of study designs such as RCTs, CCTs, and observational studies with and without control groups. Assessed quality components included: selection bias, study design, confounders, blinding, data collection methods, withdrawals and drop-outs. Strong rating is given to a study if there is no weak component score. Moderate rating is given with one weak component score. Weak rating is given with two or more component rating scores.

## Results

### Study selection

**Fig. 1:**
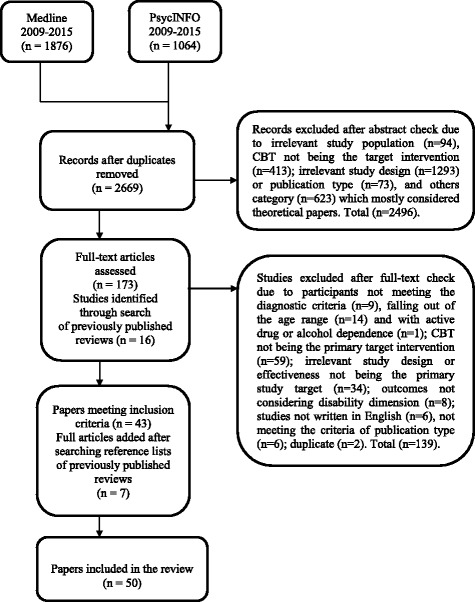
Flow diagram of the study selection process

Study selection process is presented in Fig. 1. Fifty articles were included in the review.

### Characteristics of included studies

Included studies were mostly carried out in Europe (*n* = 31), predominantly in the UK (*n* = 21) and involved 3213 participants who were recruited from in-patient and out-patient settings. Forty-four papers reported participants’ gender, with men being in majority (58.9 %). Mean age of participants ranged between 23.48 and 47.12 years. Only 14 studies reported participants’ duration of illness, the mean ranged between 3.1 and 17.7 years. Eight studies reported the number of participants’ hospitalizations, the mean ranged from 1.69 to 7.9 times. The selected studies included twenty six randomized controlled trials (RCT), eight controlled clinical trials (CCT), and sixteen cohort studies. According to the EPHPP quality assessment tool two studies (4 %) were qualified as strong, nineteen as moderate (38 %), and twenty nine (58 %) as weak. The most common reasons for the low quality of rating were missing or insufficient information regarding the selection of the study population as well as control of confounders.

Thirty-five studies considered traditional CBT interventions whereas fifteen referred to “third wave” approaches. Among these seven interventions were considered as meeting the established recovery criteria. Traditional CBT interventions were grouped according to their treatment focus, that is generic interventions (*n* = 15) or focusing on specific aspects such as hallucinations (*n* = 4), delusions (*n* = 3), negative symptoms (*n* = 2), emotions (*n* = 3), recovery (*n* = 5), suicide (*n* = 1), sleep (*n* = 1), and work (*n* = 1). Many interventions did not make a clear reference to change in beliefs, cognitive restructuring or re-evaluation of the subjective meaning of the intervention targets. Some interventions emphasized coping strategies instead [35] and others combined CBT with other treatment approaches [36–38] but overall were described as cognitive-behavioral. The interventions were delivered on an individual, group or mixed basis, or using media-delivery modes such as a computer program, mobile SMS or internet. As regards the “third wave” interventions they were grouped into generic (*n* = 1), mindfulness-based (*n* = 7), compassion-based (*n* = 1), and acceptance-based (*n* = 2) approaches, as well as person-based cognitive therapy (*n* = 1), metacognitive therapy (*n* = 1) and recovery focused approaches (*n* = 2). Most interventions were delivered on a group basis. Some of the included “third wave” studies also used traditional CBT techniques in their treatment conceptualization [39, 40].

#### Which disability domains are being addressed by current CBT approaches?

Considering conceptualization of schizophrenia disability traditional approaches focus on changing the thought content whereas “third wave” therapies focus on modifying the context and function of thoughts. We assumed that the outcomes measuring the impact of the interventions on schizophrenia disability may also differ and the results were presented separately. Outcomes measured in traditional CBT interventions are displayed in Table 1.

**Table 1:** Disability dimensions addressed by traditional CBT interventions

ICF categories	*n*	Traditional CBT									Brief ICF Core Set (*n* = 25)
		Generic (*n* = 15)	Hallucinations (*n* = 4)	Delusions (*n* = 3)	Negative symptoms (*n* = 2)	Emotions (*n* = 3)	Recovery (*n* = 5)	Suicide (*n* = 1)	Sleep (*n* = 1)	Work (*n* = 1)	
Mental functions											
*Global psychosocial functions (b122)*											x
*Temperament and personality functions (b126)*	3	x					x				
Worry (b1263)	3	x				x					
*Energy and drive (b130)*											x
Apathy (b130)	4	x			x		x				
Sleep functions (b134)	1								x		
*Attention (b140)*	3	x			x		x				x
*Psychomotor function (b147)*	1		x								
*Emotional functions (b152)*											x
Depression (b1522)	14	x	x		x		x	x			
Anxiety (b152)	8	x	x	x			x	x			
Anhedonia (b1520)	4	x			x		x				
Hostility (b1522)	2	x						x			
Flat affect (b1522)	4	x			x		x				
In general (b152)	4	x		x			x				
Anger (b152)	1					x					
*Perceptual function (b156)*	16	x	x			x	x	x	x		x
*Thought functions (b160)*	18	x	x	x		x	x	x	x		x
Suicidal ideation (b1602)	3	x						x			
*Higher-level cognitive functions (b164)*	2	x		x							x
Cognitive flexibility (b1643)	2	x		x							
Insight (b1644)	7	x	x				x				
*Alogia (b167)*	4	x			x		x				
*Experience of self and time functions (b180)*											x
Activities and participation											
*Acquiring skills (d155)*											x
*Solving problems (d175)*											x
*Carrying out daily routine (d230)*											x
*Handling stress and other psychological demands (d240)*											x
*Looking after one’s health (d570)*											x
Treatment adherence (d5702)	2	x									
Interpersonal interactions and Relationships in general (d7)	2	x					x				
*Basic interpersonal interactions (d710)*											x
*Complex interpersonal interactions (d720)*											x
Aggressive behaviour (d7202)	2	x				x					
*Family relationships (d760)*											x
*Employment in general (d850)*	2	x								x	
*Work efficiency (d850)*	2						x			x	
*Acquiring, keeping and terminating a job (d845)*											x
*Community life (d910)*											x
Personal factors											
Satisfaction in general	1	x									
Self-perception: self esteem	6	x	x				x	x			
Believes/awareness	4	x		x	x		x				
Social skills	1						x				
General interpersonal skills	1						x				
Living independently	2	x					x				
Global scores related to disability											
Negative symptoms	18	x	x		x		x	x			
Positive symptoms	16	x	x		x		x	x			
Global symptomatology	18	x	x	x	x	x	x	x	x		
General psychopathology	7	x	x	x	x	x	x	x	x		
Global disability or global functioning	10	x	x		x	x	x	x			
Social functioning	3	x	x				x				
Others											
Quality of life	7	x	x				x		x		
Wellbeing	3					x	x		x		

*n* the number of articles addressing the disability dimension, *x* considered disability dimension

Disability aspects addressed in “third wave” approaches are presented in Table 2.

**Table 2:** Disability dimensions addressed by “third wave” CBT

ICF codes	*n*	Third wave CBT							Brief ICF Core Set (*n* = 25)
		Generic (*n* = 1)	Mindfulness based (*n* = 7)	Compassion based (*n* = 1)	Acceptance based (*n* = 2)	Person-based cognitive therapy (*n* = 1)	Metacognitive therapy (*n* = 1)	Recovery (*n* = 2)	
Mental functions									
*Global psychosocial functions (b122)*									x
*Temperament and personality functions (b126)*									
Mood (b1263)	1							x	
Optimism (b1265)	1		x						
*Energy and drive functions (b130)*									x
*Attention functions (b140)*									x
*Emotional functions (b152)*									x
Depression (b1522)	7	x	x	x	x		x	x	
Anxiety (b152)	3		x		x		x		
Anhedonia (b1520)	1		x						
Hostility (b1522)	1		x						
In general	3	x	x						
Mania (b152)	1	x							
Emotional regulation (b1521)	1	x							
*Perceptual function (b156)*	4		x		x	x	x		x
*Thought functions (b160)*	3		x						x
*Higher-level cognitive functions (b164)*									x
Insight (b1644)	3	x	x		x				
*Sleep functions (b134)*									
In general (b134)	2		x						
*Experience of self and time functions (b180)*									x
Activities and participation									
*Acquiring skills (d155)*									x
*Solving problems (d175)*									x
*Carrying out daily routine (d230)*									x
*Handling stress and other psychological demands (d240)*									x
*Looking after one’s health (d570)*									x
*Basic interpersonal interactions (d710)*									x
*Complex interpersonal interactions (d720)*									x
*Family relationships (d760)*									x
*Acquiring, keeping and terminating a job (d845)*									x
*Community life (d910)*									x
Personal factors									
Perception and experience of social support	1		x						
Satisfaction in general	2		x		x				
Self-perception (in general)	1							x	
Self-perception: self esteem	1							x	
Self-perception: self-image	1							x	
Self-efficacy	1		x						
Believes/awareness	2		x	x					
Related to disability									
Negative symptoms	8	x	x	x	x		x		
Positive symptoms	7	x	x	x	x		x		
Global psychopathology	4		x		x		x		
General psychopathology	5		x	x	x		x		
Global disability or global functioning	4	x	x		x			x	
Social functioning	1	x							
Others									
Quality of life	2		x		x				
Wellbeing	2		x			x			
Mindfulness	6		x		x			x	
Acceptance	1				x				
Self-compassion	2			x				x	
Experiential avoidance	3		x	x				x	
Cognitive fusion	1							x	

*n* the number of articles addressing the disability dimension, *x* considered disability dimension

It becomes evident that both traditional and “third wave” CBT approaches measured domains of mental functions, however only studies evaluating traditional CBT interventions addressed activity and participation domains. In traditional CBT approaches measured mental functions mostly referred to thought functions (*n* = 18), perceptual functions (*n* = 16), followed by emotional functions such as depressive mood (*n* = 14) and anxiety (*n* = 8). “Third wave” interventions focused more on emotional functions such as depression (*n* = 7) rather than perceptual (*n* = 4) or thought functions (*n* = 3). In the domain of activity and participation traditional CBT addressed employment (*n* = 4), relationships with others (*n* = 4) and treatment adherence (*n* = 2). There was also a number of outcomes in both traditional and “third wave” CBT interventions that we could not directly link to the ICF categories, therefore they were grouped under personal factors, global scores related to disability and others category. Results considering these categorizations also show a high number of studies measuring the impact of interventions on schizophrenia psychopathology, which was followed by global disability and global functioning scores.

#### Are there CBT interventions that focus on personal recovery?

Seven studies were considered as focusing on personal recovery (Table 3).

**Table 3:** Characteristics of recovery focused interventions

Study (country)	Recovery	Study design	n	Intervention group	Control group	Follow up	Outcome measures	Impact on disability	Quality of rating
Farhall et al., 2009 Australia	*Recovery concept*: recovery. *Main content*: the recovery therapy intervention is a form of CBTp, which focuses on agreed recovery goals using one or more recovery therapy components such as everyday coping, working with symptoms, understanding experience of psychosis, strengthening adaptive view of self, personal/emotional issues or comorbid disorders, relapse prevention, and family or social reintegration.	RCT	94 total 45 intervention 49 control	Recovery therapy (CBTp) + TAU Individual 12–24 sessions	TAU	9 months	Primary measures: PANSS; HADS. Secondary measures: RSE; Self Report Insight Scale; LSP.	No statistically significant differences between CBTp + TAU and TAU.	Weak
Fowler et al., 2009 The UK	*Recovery concept*: social recovery. *Main content*: stage one involved formulation of the person in social recovery as well as identifying day-to-day meaningful personal goals to address motivation and hopelessness. Stage two involved identifying and working towards medium- to long-term goals and promotion of a sense of agency and addressing hopelessness, feelings of stigma and negative beliefs about self and others. Stage three involved the active promotion of social activity, work, education and leisure linked to meaningful goals, while managing symptoms of anxiety and low-level psychotic symptoms.	CCT*	77 total 35 intervention 42 control	Social Recovery Cognitive Behaviour Therapy (SRCBT) + TAU Mean of 12 sessions	TAU	No follow up	Primary measures: Time Use Survey Secondary measures: PANSS; BHS; QLS; Tertiary assessments: BDI-II; BAI; SOFAS; CAN.	No main effects of CBT treatment for any of the outcome variables for the total sample. *Global scores:* Non-affective psychosis group improved on PANSS. Non-affective psychosis group improved on constructive economic activity and structured activity (Time Use Survey).	Moderate
Grant et al., 2012 The USA	*Recovery concept*: the Recovery Movement with central features referring to goal-directed framework, personalized and person-oriented therapeutic approach highlighting the patients’ interests, assets, and strengths. *Main content*: initial sessions focused on enhancing the therapeutic relationship and stimulating patients’ interest and motivation to focus respectively on achievable goals. Impediments to goals achievement were also addressed in the later phases of the intervention.	RCT	60 total 31 intervention 29 control	Cognitive Therapy plus standard treatment (ST) Flexible	Standard treatment (ST)	6 months; 12 months; 18 months.	Primary measures: GAS. Secondary measures: SANS, SAPS.	*Mental functions:* Avolition-apathy (SANS) across the trial *Global scores:* Positive symptoms (SAPS) across the trial. Global functioning (GAS) across the trial.	Moderate
Johns et al., 2015 The UK	*Recovery concept:* recovery referred to as “living a satisfying, hopeful and contributing life even with limitations caused by the illness” and “having a sense of purpose and direction”. *Main content:* the authors described the interventions as compatible with conceptualizations of recovery. The intervention promoted psychological flexibility (a more accepting, mindful, and de-fused approach) in response to symptoms of psychosis and associated emotions/thoughts, in order to help the person act in accordance with their personal values.	Pre + post	89 total	Acceptance and Commitment Therapy Group 4 sessions, one optional telephone session	No control group	20 weeks	The Sheehan Disability Scales, HADS, AAQ-II, CFQ, SMQ.	*Mental functions*: Mood over time (HADS) *Global scores:* Functioning over time (The Sheehan Disability Scales) *Other:* Processes targeted by the intervention (AAQ-II, CFQ, SMQ).	Weak
Laithwaite et al., 2009 The UK	*Recovery concept*: recovery. *Main content*: a recovery intervention was based on the compassionate mind training. During the first module of the intervention participants were encouraged to think about their recovery beyond symptom reduction and as a journey of experience. Further modules targeted compassion with reference to working on strength, acceptance, forgiveness as well as developing the ideal friend. The last module focused on developing plans for recovery after psychosis.	Pre + post	19 total	Compassionate mind training (CMT) Group 20 sessions	No control group	6 weeks	Primary measures: SCS, OAS, SeCS, BDI-II, RSE, SIP-AD. Secondary measures: PANSS.	*Mental functions:* Depression (BDI-II) *Global scores:* General psychopathology (PANSS) *Personal factors:* Comparisons to others (SCS), self-esteem (RSE), external shame (OAS).	Weak
Study (country)	Recovery	Study design	n	Intervention group	Control group	Follow up	Outcome measures	Impact on disability	Quality of rating
Penn et al., 2011 The USA	*Recovery concept*: illness management and functional recovery. *Main content*: the program placed an emphasis on personal goal pursuit to foster optimism and self-esteem, targeted malleable factors that may enhance recovery such as residual symptoms and substance use, and enlists external social support to maximize therapeutic gains and engagement. The intervention consisted of four phases: engagement and wellness management; substance use; persistent symptoms; and functional recovery.	RCT	46 total 23 intervention 23 control	Graduated Recovery Intervention Program (GRIP) (CBT) + TAU Individual 36 sessions	TAU	3 months	Primary outcomes: QLS; RFS, MCAS; SSPA. Secondary outcomes: the PANSS; CDSS; subscales from the Scales of Psychological Well-Being; MSPSS; AUS; DUS; BEMIB.	*Activity and participation domain:* Work functioning at follow-up (RFS) Within-group analysis *Mental functions:* Depression (CDSS) across the trial *Activity and participation domain:* Extended social network (RFS) across the trial *Global scores:* Total role functioning (RFS) across the trial *Personal factors:* Social competence (MCAS) across the trial	Weak
Williams et al., 2014 The UK	*Recovery concept*: the recovery model described as building a meaningful and satisfying life defined by the person themselves, focusing upon strengths and wellness not illness and pathology, a sense of hope, and possibility of change, promotion of self-management and personal identity (not patient identity), the therapeutic relationship being one of partnership not “expert-patient”; and encouragement of group members to help each other in recovery. *Main content*: The intervention was delivered in five modules. The first one focused on engagement and treatment preparation, module two on individual analysis of the person and schizophrenia, module three understanding and managing positive symptoms, module four maximizing mental health and module five reviews of personal aims and goals, reinforcement of protective factors, development of a detailed relapse recognition and staying well plan as well as discussion of future directions.	CCT	47 total 30 intervention 17 control	Cognitive-behavioural therapy Individual and group 35 planned sessions	TAU	No follow up	SAPS, SANS, PSYRATS, DASS, IIP.	*Mental functions*: Delusions (SAPS) Hallucinations (SAPS) Affective flattening (SANS) Alogia (SANS) Anhedonia (SANS) Avolition (SANS) Depression (DASS) Anxiety (DASS) Overall interpersonal problems (social inhibition and self-sacrifice) (IIP)	Moderate

*CCT* Clinical Controlled Trial, CCT* Clinical Controlled Trial (EPHPP criteria regarding RCTs where the allocation method is not described or allocation is transparent before assignment), *Pre + post* Cohort (one group pre + post (before and after)), *RCT* Randomized Controlled Trial, *TAU* Treatment as usual, *n* number of participants, *PANSS* Positive and Negative Syndrome scale, *HADS* The Hospital Anxiety and Depression Scale, *RSE* Rosenberg Self-Esteem scale, *LSP* the Life Skills Profile, *BHS* Beck Hopelessness Scale, *QLS* Quality of Life Scale, *BDI-II* the Beck Depression Inventory, *BAI* the Beck Anxiety Inventory, *SOFAS* the Social and Occupational Functioning Assessment Scale, *CAN* the Camberwell Assessment of Needs, *GAS* The Global Assessment Scale, *SANS* Scale for the Assessment of Negative Symptoms, *SAPS* The Scale for the Assessment of Positive Symptoms, *AAQ-II* The Acceptance and Action Questionnaire, *CFQ* The Cognitive Fusion Questionnaire, *SMQ* The Southampton Mindfulness Questionnaire, *SCS* Social Comparison Scale, *OAS* The Other as Shamer Scale, *SeCS* Self-Compassion Scale, *SIP-AD* The Self-Image Profile for Adults, *RFS* The Role Functioning Scale, *MCAS* The Multnomah Community Ability Scale, *SSPA* The Social Skills Performance Assessment, *CDSS* the Calgary Depression Scale for Schizophrenia, *MSPSS* The Multidimensional Scale of Perceived Social Support, *AUS* the Alcohol Use Scale, *DUS* Drug Use Scale, *BEMIB* The Brief Evaluation of Medication Influence and Beliefs, *PSYRATS* the Psychotic Symptom Rating Scales, *DASS* Depression Anxiety Stress Scale, *IIP* The Inventory of Interpersonal Problems

Selected interventions varied with regards to the recovery concept. It ranged from interventions explicitly referring to the Recovery Movement [41] or recovery definitions that resemble the spirit and goals of the recovery paradigm [42, 43] to other concepts such as social recovery [36] or functional recovery [37]. Interventions also differed with regards to their content as many studies integrated recovery approach with a wide variety of already existing therapeutic concepts and strategies. Identifying and working towards meaningful personal goals seemed to be a core element of many recovery-focused approaches.

#### What is the effectiveness of CBT interventions focusing on recovery?

The impact of recovery-focused interventions on disability domains was mostly pronounced in mental functions, namely emotional functions – depression [37, 43, 44] anxiety [43], mood [42], affective flattening, and anhedonia [43]. Improvements were also reported in the domain of energy and drive functions (avolition) [41, 43], perceptual functions (hallucinations), thought functions (delusions), language functions (alogia) and interpersonal problems [43]. Global scores related to disability also showed improvements in aspects such as global psychopathology [36], general psychopathology [44], and positive symptoms [41].

Regarding the domain of activity and participation improvements were reported in work functioning at follow-up and participation in extended social network relationships across the trial [37]. Global scores of disability and functioning showed improved functioning [37, 41, 42] as well as hours spent on economic and structured activity [36].

The above results have to be interpreted with caution as four of the studies [37, 42, 44, 45] were rated as weak with regards to the risk of bias, whereas three [36, 41, 43] were given a moderate rating.

## Discussion

In the present systematic review we provide a comprehensive overview on disability domains considered by CBT interventions in schizophrenia using the ICF as reference framework. We also examined whether there are any CBT interventions focusing on personal recovery and the impact of these interventions on disability domains. We included 35 studies evaluating traditional CBT interventions and 15 evaluating “third wave” approaches, 7 of them met our inclusion criteria of personal recovery. Traditional CBT interventions addressed more disability domains than “third wave” therapies, however in both approaches there was a strong emphasis on mental functions reflecting schizophrenia symptoms. Recovery-focused interventions differed in the degree of clarity with regards to the recovery concept. These studies show significant impact on emotional functions, negative symptoms, schizophrenia psychopathology, work functioning, participation in extended social network relationships, global disability, functioning and hours spent on economic and structured activities. However, only three recovery-focused studies were rated as fair regarding the risk of bias.

All studies included in this review have a strong focus on mental functions, especially perceptual functions, thought functions and depressive mood, and fail to measure the impact of their interventions on a broad range of activity and participation domains. However, in a recently published systematic review on psychosocial difficulties in schizophrenia [8] the proportion of reported mental functions against the activity and participation domain is comparable. Results refereeing to mental functions are in line with those reported by Świtaj et al. [8] where the most extensively studied were cognitive (27 %) and emotional functions (27 %). This resembles the core aim of many CBT interventions, i.e. targeting the distress resulting from psychotic symptoms. As reported by Jones et al. [16] outcomes in CBT for psychosis are often defined in terms of the reduction in hallucinatory and delusional experience instead of eliciting emotional and behavioral changes, however our findings also indicate the strong emphasis on outcomes related to depressive mood. This supports the stance on commonality of affective disorders in psychosis and its contribution to the suffering caused by the illness and exacerbation of deficits in psychosocial functioning often preceding attempted and completed suicide [46, 47].

With regards to activity and participation the impact of traditional CBT interventions mostly revolved around the area of relationships with others and employment which is also in line with Świtaj et al. [8] results. In studies evaluating “third wave” CBT no activity and participation domain was included as an outcome but studies reported on global scores related to disability, functioning and social functioning. In terms of treatment conceptualization “third wave” approaches differ from the traditional CBT interventions by deemphasizing the importance of changing the content and frequency of cognition while focusing on mindfulness and acceptance processes [48]. However as indicated by Khoury et al. [28] targeting these processes among people diagnosed with schizophrenia spectrum disorders later translates into improvement of symptoms, functioning and quality of life. This might explain why the area of activity and participation was not the target of “third wave” approaches.

Our results indicate a mismatch between what is targeted in CBT for schizophrenia and the scope of disabilities experienced by persons with schizophrenia in daily life. In a recent qualitative study it was shown that users’ perception of psychosocial difficulties or disability domains, revolve around the activity and participation areas such problems in relationships or finding and keeping work and place to live. Many of the indicated psychosocial difficulties also considered personal factors such as problems with self-esteem or environmental factors e.g. experience of stigma or frustrations with mental health services. Interestingly users’ views of the domain of mental functions refer to emotional functions for example feeling fear or despair but not to impairments of thought or perceptual functions [49]. This may indicate that when human experience is considered these areas of functioning seem to be less prominent.

Several reasons might explain the strong focus on mental functions. The focus of traditional CBT interventions is mostly on symptoms alleviation rather than functioning, whether by directly targeting the psychotic symptoms or distress related with them. The underlying hypothesis behind that is the assumption that symptom alleviation will automatically translate in improvement in functioning in general, what might explain why measures of global disability, functioning or social functioning are included while specific functioning domains related to activity and participation are missing. However, as shown by Wykes et al. [17] improvements in positive symptoms were correlated with improvements in negative symptoms but did not quite reach significance with regards to improved global functioning among people with schizophrenia spectrum disorders. Instead improvements in negative symptoms were indeed significantly correlated with better functioning and improved mood. The majority of traditional CBT interventions included in this review targeted a wide spectrum of symptoms including negative symptoms and mood while “third wave” interventions targeted emotional distress arising from psychotic symptoms. Having in mind the recent works shading light on the activity and participation outcomes important for persons with schizophrenia, it would be highly relevant if studies would indeed examine the effectiveness of their interventions in these areas.

Although expectations of people with lived experience of schizophrenia revolve around the recovery paradigm, we have identified few CBT interventions focusing on personal recovery. Studies included in our review reflect the diversity of existing recovery definitions. They also varied in their scope, however identifying and working towards meaningful personal goals seemed to be a core element of many recovery-focused approaches. With regards to the targeted disability dimensions the included recovery interventions addressed relatively wide scope of both mental functions and activity and participation domains. Interestingly they did not extensively focused on perceptual or thought functions but targeted disability aspects that mostly revolve around negative symptoms and emotional functions. In the area of activity and participation they targeted relationships, and employment. This points out that these interventions come closer to the desired broad perspective than traditional CBT interventions due to targeting personal recovery process but also using objectives measures of negative symptoms which were shown to be related with increased functioning and mood.

The evidence on effectiveness of these interventions is inconclusive, due to the fact that many addressed outcomes considered global scores of disability and functioning and that four of the seven included studies had weak rating regarding study quality. Studies of good methodological quality including as outcomes not global functioning but the specific functioning domains relevant to persons with schizophrenia are therefore needed.

This review should be interpreted considering several limitations. Firstly, we only analyzed papers that were published in English and used two databases. Nevertheless, to complement the search we checked reference lists of other systematic reviews. Secondly, we set the time limit 2009–2015 for the selected studies, therefore it is possible we did not consider some relevant papers that were published before this time frame. Thirdly, we cannot generalize the findings as majority of studies came from high income countries and disabilities in schizophrenia may depend on the economic, political and cultural context [8]. Fourthly, despite the existence of guidelines for developing recovery-oriented services we felt that the implications for clinical practice were not clear [50, 51]. Therefore, we used the CHIME personal recovery framework as it provides useful information regarding the key recovery process to be potentially targeted by the interventions [12, 52]. Another limitation of the study is that integrative metacognitive models [53, 54], which have been specifically designed to target subjective recovery were not considered in our review. Further studies should evaluate the impact of such interventions on schizophrenia disability.

## Conclusions

Despite the growing need of shifting the focus from symptom-oriented approaches to treatment conceptualizations that support real-world functioning of service users, our study indicate that traditional and “third wave” CBT interventions mostly focus on aspects of disability that relate to mental functions. There are also few interventions that focus on personal recovery, however they seem to be a promising treatment approach as they target disability from a broader perspective including activity and participation domains. Despite the limited evidence of their effectiveness it might be valuable to explore further possibilities of developing recovery-oriented CBT interventions as they reflect users’ views of recovery and target disability outcomes that in previous studies have been shown to improve mood and functioning. Perhaps the best practices from both traditional and “third wave” approaches could be combined in order to maximize their therapeutic potential in terms of what matters to persons with schizophrenia.

## Abbreviations

ACT, acceptance and commitment therapy; CBT, cognitive – behavioral interventions/therapy; CBTp, cognitive-behavioral therapy for psychosis; CCT, Clinical Controlled Trial; CHIME, connectedness, hope and optimism about the future, identity, meaning in life, empowerment; DBT, dialectical behavior therapy; DSM-IV, American Psychiatric Association Diagnostic and Statistical Manual of Mental Disorders, fourth edition; EPHPP, Effective Public Health Practice Project Quality assessment tool for quantitative studies; FAP, functional analytic psychotherapy; IBCT, integrative couple therapy; ICD-10, the International Classification of Diseases, tenth edition; ICF, International Classification of Functioning, Disability and Health; MBCT, mindfulness-based cognitive therapy; MCT, metacognitive therapy; RCT, Randomized Controlled Trial

## Additional file

Additional file 1: Search strategies for MEDLINE and PsycINFO. (DOCX 14 kb)
