# Supplementary material for: Disability and recovery in schizophrenia: a systematic review of cognitive behavioral therapy interventions
Source: BMC Psychiatry. 2016 Jul 11;16:228. doi: 10.1186/s12888-016-0912-8 (PMC4940955; doi:10.1186/s12888-016-0912-8)
Supplement: Additional file 1: — Search strategies for MEDLINE and PsycINFO. (DOCX 14 kb) [file 12888_2016_912_MOESM1_ESM.docx]

**Additional file 1**

**Final Search Strategy Medline**

1. Exp Schizophrenia/ OR schizophren*

2. cognitive and behavior and therapy

3. cogniti* and (technique* or therap* or restructur* or challeng*)

4. attribution* or (self and (instruct* or management* or attribution*)

5. ret OR (rational AND emotive)

6. Cognitive-therapy/ OR CBT OR Cognitive Behavioural Therapy

7. mindfulness OR “third wave” OR mindfulness-based therapies OR compassion OR acceptance

8. therap* and (acceptance* or commitment*)

9. (thought* and suppress*) or rumination

10. 2 OR 3OR 4 OR 5 OR 6 OR 7 OR 8 OR 9

11. 1 AND 10

12. primary prevention studies OR clinical trial, phase 1/ OR clinical trial, phase 2/ OR ecologic studies OR case reports/ OR case series OR exp Cross-Sectional Studies/ OR Qualitative Research/ exp Review/ OR Literature review as topic/ OR evidence based review.ti.

13. 11 NOT 12

14. Child OR Adolescent

15. 13 NOT 14

16. mouse OR mice OR rat OR rats OR rabbit OR rabbits OR guinea?pig* OR animal model*

17. 16 NOT 18

18. Date of Publication: 2009/01/01-2015/12/31; English Language; Publication Type: Journal Article

**Final Search Strategy PsycINFO**

1. Exp Schizophrenia/ OR schizophren*

2. Cognitive behavior Therapy/ OR mindfulness/ OR Acceptance and commitment therapy/ OR CBT OR Cognitive Behaviour Therapy OR restructur* OR attribution* OR ACT OR mindfulness OR third wave OR compassion OR emotional regulation OR sympathy OR acceptance

3. 1 AND 2

4. primary prevention studies OR phase I studies OR phase II studies OR ecologic studies OR Cross-Sectional* OR "Surveys" OR Case Report OR case series OR economic evaluations OR qualitative* OR Literature Review/ OR systematic review

5. 3 NOT 4

6. Published Date: 2009/01/01-2015/12/31; Publication Type: Peer Reviewed Journal; Population Group: Human

7. Child and adolescents

8. 6 NOT 7
